# Mean Platelet Volume and Gestational Diabetes Mellitus: A Systematic Review and Meta-Analysis

**DOI:** 10.1155/2018/1985026

**Published:** 2018-05-02

**Authors:** Zhongwei Zhou, Hongmei Chen, Mingzhong Sun, Huixiang Ju

**Affiliations:** Department of Clinical Laboratory, Affiliated Yancheng Hospital, School of Medicine, Southeast University, Yancheng, Jiangsu 224001, China

## Abstract

**Aim:**

To evaluate the association between mean platelet volume (MPV) and gestational diabetes mellitus (GDM).

**Methods:**

A systematic literature search was performed in PubMed, EMBASE, Web of Science, and The Cochrane Library up to 4 September 2017. Pooled standardized mean differences (SMD) and 95% confidence interval (CI) were calculated using a random-effect model.

**Results:**

Nineteen studies comprising 1361 GDM patients and 1911 normal pregnant women were included. MPV was increased in GDM patients when compared with healthy pregnant women (SMD: 0.79; 95% CI: 0.43–1.16; *P* < 0.001). Subgroup analyses revealed that such trend was consistent in the third-trimester (SMD: 1.35; 95% CI: 0.72–1.98), Turkish (SMD: 0.81; 95% CI: 0.43–1.19), and Italian (SMD: 2.78; 95% CI: 2.22–3.34) patients with GDM and the patients diagnosed based on Carpenter and Coustan criteria (SMD: 1.04; 95% CI: 0.42–1.65). Significantly higher MPV also were observed within cross-sectional studies (SMD: 0.99; 95% CI: 0.49–1.49). Remarkable between-study heterogeneity and potential publication bias were observed in this meta-analysis; however, sensitivity analysis indicated that the results were not unduly influenced by any single study.

**Conclusions:**

GDM patients are accompanied by increased MPV, strengthening the clinical evidence that MPV may be a predictive marker for GDM.

## 1. Introduction

Gestational diabetes mellitus (GDM), one of the most prevalent pregnancy complications, is defined as varying degrees of impaired glucose intolerance that is initially recognized in pregnancy [1]. According to different definition and criteria, GDM affects 4–18% pregnant women [2]. Although glucose intolerance may return to normal after pregnancy in most women with GDM, a certain proportion of pregnant women will develop into or are at greatest risk for progression to type 2 diabetes mellitus (T2DM) [3, 4]. And like T2DM, GDM patients generally have different levels of insulin resistance and chronic low-grade inflammation, which trigger vascular injury and dysfunction and subsequent platelet activation [5, 6]. Therefore, the level of platelet activation may be associated with severity of GDM.

Mean platelet volume (MPV), an easily and inexpensive parameter derived from routine blood counts, is usually used to evaluate platelet morphology and can be used as an indicator of platelet activity [7]. Elevated MPV has been demonstrated to be related to cardiovascular diseases and its risk factors such as T2DM, hypertension, and nonalcoholic fatty liver disease (NAFLD) [8–11]. Recently, a substantial number of studies measured MPV levels in women with GDM to evaluate whether it can be used as an indicator to monitor and assess the development of GDM. However, studies of the association between MPV and GDM yielded inconsistent findings. Some studies observed that GDM patients had significantly increased MPV compared with healthy pregnant women [12, 13], whereas other studies reported no association between MPV and GDM [14, 15]. Furthermore, some groups found decreased MPV values in patients with GDM [16, 17]. In light of these inconsistent findings, we undertook a meta-analysis to provide a more comprehensive conclusion of the association between MPV and GDM.

## 2. Methods

### 2.1. Search Strategy

A systematic search on literature was performed on electronic databases including PubMed, EMBASE, Web of Science, and The Cochrane Library up until 4 September 2017. The search terms included (“gestational diabetes mellitus” OR gestational diabetes OR “GDM”) AND (“mean platelet volume” OR MPV). In addition, the bibliographies from these relevant articles were also manually searched for additional eligible studies.

### 2.2. Study Selection

Studies were considered eligible if they met the following criteria: (1) studies compared the MPV between GDM patients and healthy pregnant women with normal glucose tolerance (NGT), (2) case and control subjects all did not have a previous history of diabetes or present pregnant complications, or (3) studies were published in English or Chinese. Studies were excluded if they were reviews, editorials, letters to the editor, case reports, conference abstracts, or studies on animals or cell lines.

### 2.3. Data Extraction and Quality Assessment

Two investigators (Zhongwei Zhou and Hongmei Chen) independently reviewed all identified studies and extracted the data using a predefined form and confirmed by a third reviewer (Huixiang Ju). Disagreement was resolved by discussion among all researchers. The following information was abstracted from each eligible study: the first author's name, year of publication, study location, study design, trimester of MPV measurement, average age and body mass index (BMI) of GDM patients, diagnosis criteria of GDM, sample size of the case and control group, and mean and standard deviation (SD) of MPV. If studies did not offer mean and SD of MPV, the corresponding authors were contacted. When the request was not responded, transformations were made by standard formulas.

The quality of the study was evaluated using a modified criteria based on the Newcastle-Ottawa Quality Assessment Scale (NOS) for observational studies suggested by van Dijk et al. [18], which was modified to accommodate the topic of this review. A study that met 7 or more points would be considered as a high-quality study.

### 2.4. Statistical Analysis

Standardized mean differences (SMD) and 95% confidence interval (CI) in MPV between GDM patients and controls were calculated and estimated for each study. A random-effect model was chosen for pooling of data. This is because if there is a significant heterogeneity between studies, the random-effect model would be more conservative than the fixed-effect model [19]. Heterogeneity across included studies was assessed using the *I*^2^ index, and an *I*^2^ index of 25%, 50%, and 75% would indicate small, moderate, and high heterogeneity, respectively [20]. To explore the potential source of heterogeneity, subgroup analysis was carried out by the trimester of MPV measurement, study location, and study design.

Sensitivity analysis was performed to evaluate the influence of each study on the pooled measures by omitting one single study in each turn and recalculating the pooled SMD for the remainders. Publication bias was evaluated by inspection of funnel plots and Egger's test.

All analyses were performed using Stata 14.0 (StataCorp LP, College Station, TX, USA), and *P* < 0.05 was considered to be statistically significant.

## 3. Results

### 3.1. Study Selection and Study Characteristics

**Figure 1:**
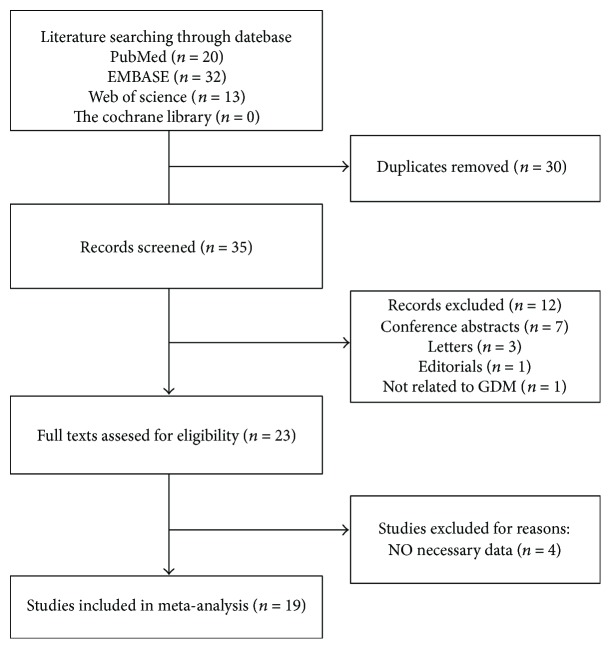
Flow chart of the study selection process.

The electronic database search of PubMed, EMBASE, Web of Science, and The Cochrane Library yielded a total of 65 records. After removing duplicates and reading the titles and abstracts, 23 appropriate articles were identified for full-text scrutiny. The 4 articles were further excluded for lack of necessary data. Finally, 19 studies (20 results) met the criteria to be included in the present meta-analysis [12–17, 21–33], and a flowchart showed the selection process (Figure 1).

The 19 included studies were published from 2005 to 2017 covering 1361 GDM patients and 1911 normal pregnant women. The main characteristics of these studies included in the present meta-analysis are presented in Table 1. Among these, twelve cross-sectional and seven case-control studies (8 results) were included. Fifteen studies were carried out in Turkey, three in China, and one in Italy. Several different criteria were used to define GDM, and among them, Carpenter and Coustan (C&C) criteria were used in nine studies, National Diabetes Data Group (NDDG) in three studies, and American Diabetes Association (ADA) in five studies. Nine studies measured MPV values during the second trimester and the same number during the third trimester. The results of quality evaluation showed that the mean score across included studies was 5.8. Eight studies were scored greater than or equal to 7 out of 9 which were considered as high-quality studies. However, two studies, Maconi et al. [25] and Sahbaz et al. [13], were graded 2 and 3, respectively.

### 3.2. Main Association of MPV with GDM

**Figure 2:**
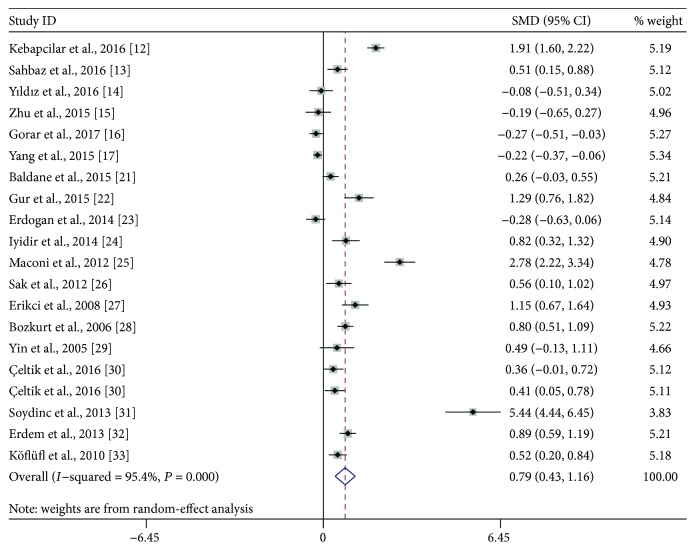
Overall meta-analysis of mean platelet volume in gestational diabetes mellitus patients compared with healthy pregnant women. SMD: standardized mean differences; CI: confidence interval.

We performed a random-effect meta-analysis on the extracted 19 studies. The results showed that MPV values were significantly increased in GDM patients when compared with healthy pregnant women (Figure 2, SMD: 0.79; 95% CI: 0.43, 1.16; *P* < 0.001). Sensitivity analysis showed that no individual study significantly influenced the difference on MPV values between GDM patients and healthy pregnant women. However, significant and high-level heterogeneity among studies was found in this meta-analysis (*I*^2^ = 95.4, *P* < 0.001).

### 3.3. Subgroup Analyses

**Figure 3:**
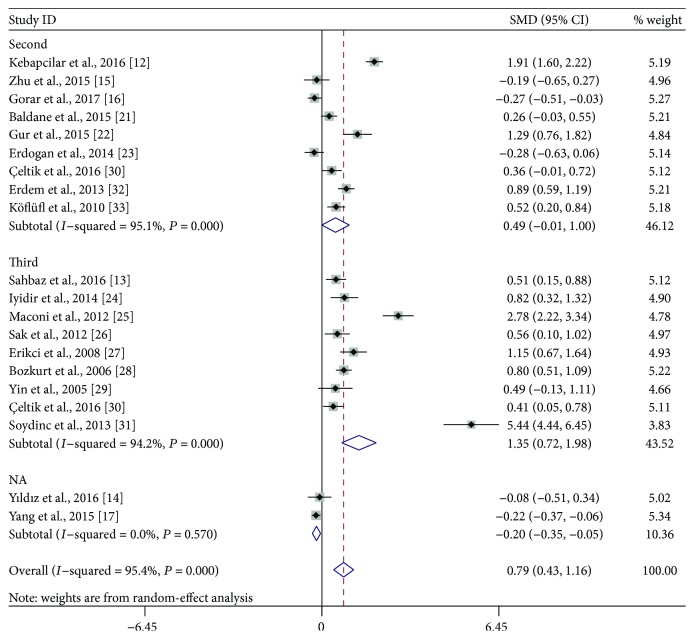
Subgroup analysis of mean platelet volume (MPV) in gestational diabetes mellitus patients compared with healthy pregnant women when stratified by the trimester of MPV measurement. SMD: standardized mean differences; CI: confidence interval; NA: not available.

**Figure 4:**
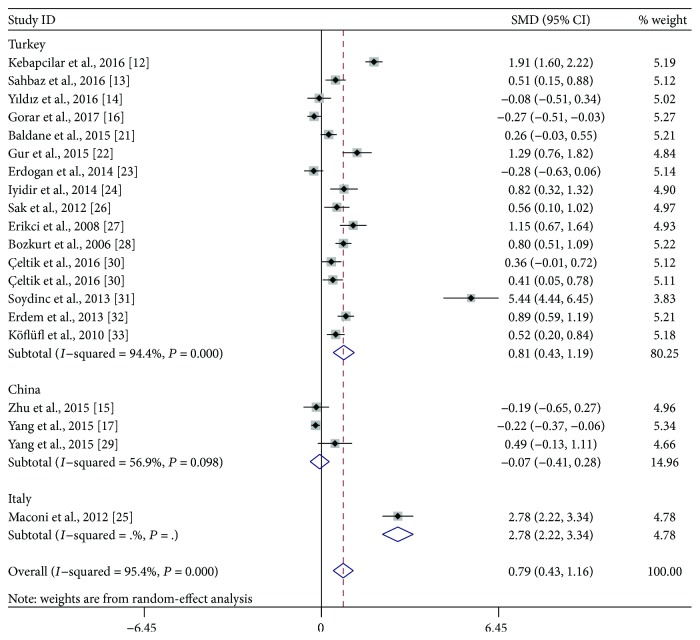
Subgroup analysis of mean platelet volume (MPV) in gestational diabetes mellitus patients compared with healthy pregnant women when stratified by study location. SMD: standardized mean differences; CI: confidence interval.

**Figure 5:**
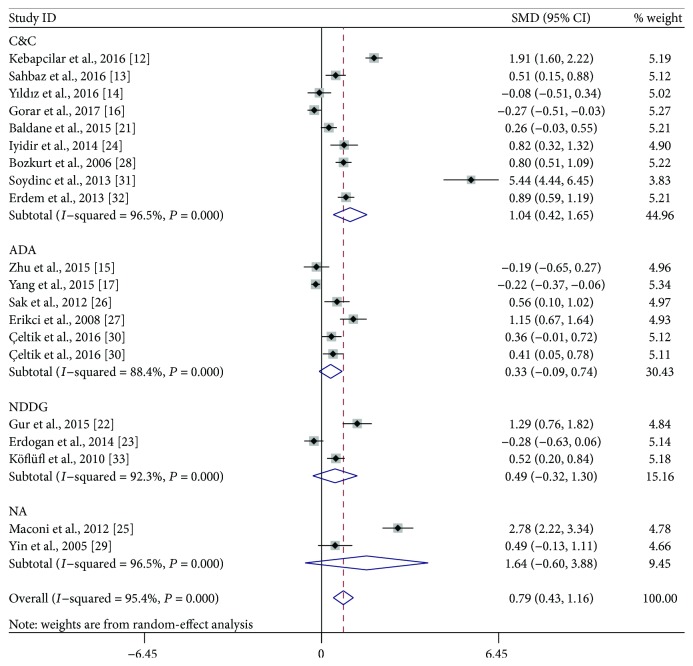
Subgroup analysis of mean platelet volume (MPV) in gestational diabetes mellitus patients compared with healthy pregnant women when stratified by the defined criteria of GDM. SMD: standardized mean differences; CI: confidence interval; NA: not available.

**Figure 6:**
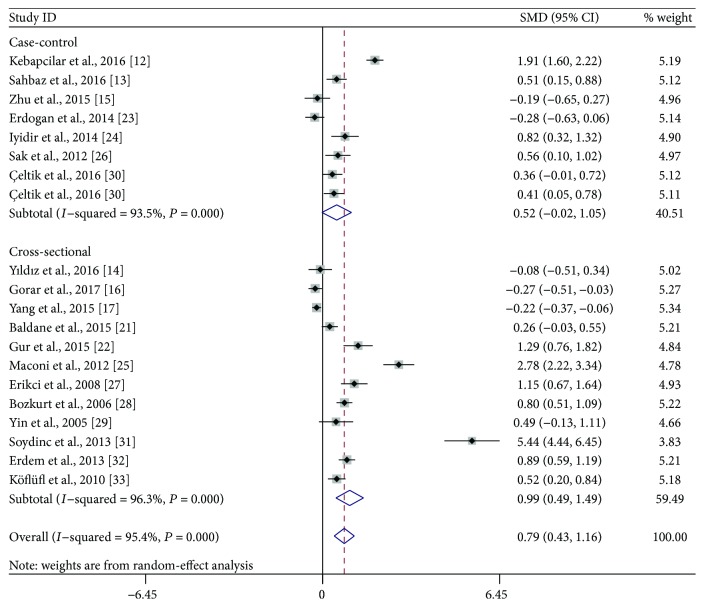
Subgroup analysis of mean platelet volume (MPV) in gestational diabetes mellitus patients compared with healthy pregnant women when stratified by study design. SMD: standardized mean differences; CI: confidence interval.

Subgroup analyses were performed based on the trimester of MPV measurement, study location, the defined criteria of GDM, and study design. As shown in Figure 3, stratified analyses indicated that the third-trimester patients had significantly higher MPV than did healthy pregnant women (SMD: 1.35; 95% CI: 0.72–1.98), while the difference did not reach statistical significance during the second trimester (SMD: 0.49; 95% CI: −0.01–1.00). When the studies were classified to three subgroups according to study location, both Turkish (SMD: 0.81; 95% CI: 0.43–1.19) and Italian (SMD: 2.78; 95% CI: 2.22–3.34) patients had significantly higher MPV compared with the control, but the difference was not observed between Chinese women with and without GDM (SMD: −0.07; 95% CI: −0.41-0.28) (Figure 4). When stratifying by the defined criteria of GDM, patients defined by C&C criteria had significantly higher MPV compared with the control (SMD: 1.04; 95% CI: 0.42–1.65), but those defined by other criteria had not (Figure 5). The significant difference was also observed in the subgroup of cross-sectional studies (SMD: 0.99; 95% CI: 0.49–1.49), but not within the subgroup of case-control studies (SMD: 0.52; 95% CI: −0.02–1.05) (Figure 6).

### 3.4. Publication Bias

**Figure 7:**
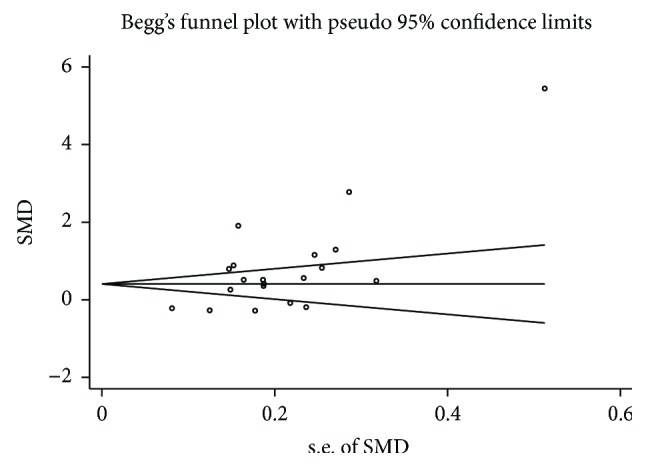
Begg's funnel plot of included studies for potential publication bias between gestational diabetes mellitus patients and healthy pregnant women. SMD: standardized mean differences; s.e.: standard error.

Visual inspection of funnel plots showed asymmetry in this meta-analysis (Figure 7). Egger's test further showed that there was potential publication bias in this meta-analysis (*P* = 0.004).

## 4. Discussion

This meta-analysis demonstrated that MPV was significantly increased in GDM patients compared with healthy pregnant women overall. Although potential publication bias might exist in the included studies, sensitivity analysis indicated that the results were not unduly influenced by any single study. These findings suggest that women with GDM may be accompanied by increased MPV levels. To the best of our knowledge, this is the first meta-analysis on this subject, which provides clinical evidence that MPV may be a predictive marker for GDM.

In the meta-analysis, subgroup analysis was used to analyze the potential factors contributing to heterogeneity and obtain further information from different subpopulations. When stratifying by the trimester of MPV measurement, we observed significantly increased MPV in GDM patients in the third trimester. Significantly higher MPV was also observed in Turkish and Italian women with GDM and the subgroups of cross-sectional studies and C&C criteria defined for GDM patients. These results suggest that there may be varying levels of MPV in different stages of pregnancy and GDM patients with different ethnic backgrounds, and study design and the diagnostic criteria of GDM might also influence the results of MPV. Although subgroup analyses were carried out to explore some potential sources, we still found high levels of heterogeneity in all the subgroups but studies with the Chinese patient group in which heterogeneity was moderately reduced. As data on some potential confounders such as BMI, insulin resistance index, and lifestyle are limited in the eligible studies included in the meta-analysis, which prevented us from further analyzing whether these factors were confounders affecting the outcome of this meta-analysis. Therefore, the results of this meta-analysis should be cautiously interpreted.

The pathophysiologic mechanism for increased MPV observed in GDM is not yet fully elucidated. However, several plausible explanations may account for their relationship. It has been suggested that insulin resistance is a major determinant of platelet activation which can be measured by MPV [6, 34]. Normal pregnancy is characterized by physiological insulin resistance that begins in the second trimester and peaks in the third trimester, which causes increased insulin secretion [35]. GDM is the result of increased insulin production which cannot compensate for the increased insulin resistance [36]. In this study, we found that although GDM patients had higher MPV levels than those in the control in the second trimester, the difference did not reach statistical significance, while such difference was dramatically significant during the third trimester. The diverse results of MPV levels just matched the level of insulin resistance in different trimesters. In support of this theory, two studies [12, 21] included in the present meta-analysis demonstrated that there was a significant positive correlation between MPV and homoeostasis model assessment of insulin resistance (HOMA-IR). Another plausible mechanism is the intimate connection between platelet activation and inflammation. Platelet-derived inflammatory mediators, such as soluble CD40 ligand and CD36, were considered to have significant effects on the release of cytokines and chemokines and the enhancement of the inflammatory process when the platelet is activated [37]. On the other hand, there is evidence that leukocytes can release the platelet-activating factor to induce platelet activation [38]. And it has been shown that GDM is a chronic inflammatory condition, with increased proinflammatory cytokines like TNF-*α*, IL-6, CRP, IL-1*β*, and IL-18 [5, 39]. In a prospective study, increased early pregnancy leukocyte count was shown to be independently associated with the risk of GDM [40]. In this sense, MPV may serve as an indicator of chronic inflammatory status of GDM.

There are several limitations in this meta-analysis. First, all the included studies were observational; therefore, a causal link between MPV and GDM cannot be established. Second, as most of the studies included in this meta-analysis were carried out in Turkey (15 out of 19), the pooled outcome of the meta-analysis might not be representative of the total global population. Third, the asymmetric funnel plot suggested that the potential publication bias may be present among studies, so the significant differences of MPV between GDM patients and healthy pregnant women may be overestimated. However, sensitivity analysis indicated that no individual study significantly influenced the difference on MPV levels between patients and controls. In addition, it should be acknowledged that the asymmetric funnel plot is not always created by publication bias but can also be caused by low-quality studies and significant heterogeneity among studies [41, 42]. Most recently, Zwetsloot et al. [43] revealed that the funnel plot of the SMD plotted is susceptible to distortion, resulting in overestimation in publication bias assessments. Finally, due to a lack of appropriate quality-assessment tool for observational studies, our assessment of study quality was based on the modified NOS, which may lead to arbitrary results [44]. But the quality score was not used in the meta-analyses, such as subgroup analyses, as we thought that it may be better suited to assessing different aspects of methodology of a study in an independent manner.

## 5. Conclusion

In conclusion, this meta-analysis demonstrated that GDM patients are accompanied by increased MPV, which suggests MPV may be used as an indicator to monitor and evaluate the development of GDM. However, the results should be cautiously interpreted because of the potential publication bias and substantial between-study heterogeneity, and further prospective, multicenter cohort studies are required to confirm these findings.

## Figures and Tables

**Table 1:** Main characteristics of the studies included in this meta-analysis.

References	Study location	Study design	Case group		Control group		MPV measurement trimester	Average age of GDM patients (years)	Average BMI of GDM patients (kg/m^2^)	GDM criteria	Quality score
			Sample size	MPV levels	Sample size	MPV levels					
Kebapcilar et al., 2016 [12]	Turkey	Case-control	101	9.16 ± 1.04	138	7.42 ± 0.80	Second	26.1	26.9	C&C	8
Sahbaz et al., 2016 [13]	Turkey	Case-control	59	8.93 ± 0.98	60	8.39 ± 1.11	Third	31.4	26.3	C&C	3
Yıldız et al., 2016 [14]	Turkey	Cross-sectional	53	9.98 ± 1.22	35	10.05 ± 1.15	NA	31.5	27.9	C&C	7
Zhu et al., 2015 [15]	China	Case-control	36	10.76 ± 2.02	36	11.07 ± 0.90	Second	31.2	NA	ADA	8
Gorar et al., 2017 [16]	Turkey	Cross-sectional	110	10.4 ± 0.74	159	10.6 ± 0.74	Second	33.0	NA	C&C	4
Yang et al., 2015 [17]	China	Cross-sectional	302	9.0 ± 1.47	310	9.3 ± 1.41	NA	30.6	21.2	ADA	7
Baldane et al., 2015 [21]	Turkey	Cross-sectional	114	10.2 ± 1.05	76	9.9 ± 1.27	Second	29.4	NA	C&C	5
Gur et al., 2015 [22]	Turkey	Cross-sectional	16	10.9 ± 1.0	167	9.6 ± 1.0	Second	29.0	27.1	NDDG	8
Erdogan et al., 2014 [23]	Turkey	Case-control	68	10.5 ± 2.94	61	11.2 ± 1.14	Second	31.8	NA	NDDG	5
Iyidir et al., 2014 [24]	Turkey	Case-control	30	8.8 ± 1.0	38	8.1 ± 0.70	Third	33.7	NA	C&C	8
Maconi et al., 2012 [25]	Italy	Cross-sectional	25	11.9 ± 1.90	100	8.3 ± 1.10	Third	31.4	NA	NA	2
Sak et al., 2012 [26]	Turkey	Case-control	42	8.9 ± 1.90	35	7.8 ± 2.0	Third	31.2	28.6	ADA	6
Erikci et al., 2008 [27]	Turkey	Cross-sectional	34	9.3 ± 1.45	45	8.1 ± 0.66	Third	29.5	NA	ADA	4
Bozkurt et al., 2006 [28]	Turkey	Cross-sectional	100	9.4 ± 1.6	100	8.3 ± 1.1	Third	31	NA	C&C	5
Yin et al., 2005 [29]	China	Cross-sectional	21	9.5 ± 1.6	20	8.7 ± 1.6	Third	27.1	NA	NA	5
Çeltik et al., 2016 [30]	Turkey	Case-control	105	8.66 ± 1.15	40	8.27 ± 0.92	Second	33.4	27.7	ADA	7
Çeltik et al., 2016 [30]	Turkey	Case-control	105	9.59 ± 1.34	40	8.95 ± 1.98	Third	33.4	27.7	ADA	
Soydinc et al., 2013 [31]	Turkey	Cross-sectional	42	9.28 ± 0.35	33	7.71 ± 0.19	Third	32.5	28.5	C&C	8
Erdem et al., 2013 [32]	Turkey	Cross-sectional	58	9.43 ± 1.09	219	8.53 ± 0.99	Second	30.1	NA	C&C	6
Köflüfl et al., 2010 [33]	Turkey	Cross-sectional	45	8.67 ± 1.43	239	8.19 ± 0.85	Second	31.2	NA	NDDG	5

MPV: mean platelet volume; GDM: gestational diabetes mellitus; BMI: body mass index; C&C: Carpenter and Coustan; NDDG: National Diabetes Data Group; ADA: American Diabetes Association; NA: not available.
